# Phosphoproteome Microarray Analysis of Extracellular Particles as a Tool to Explore Novel Biomarker Candidates for Alzheimer’s Disease

**DOI:** 10.3390/ijms25031584

**Published:** 2024-01-27

**Authors:** Tânia Soares Martins, Steven Pelech, Maria Ferreira, Beatriz Pinho, Kevin Leandro, Luís Pereira de Almeida, Benedict Breitling, Niels Hansen, Hermann Esselmann, Jens Wiltfang, Odete A. B. da Cruz e Silva, Ana Gabriela Henriques

**Affiliations:** 1 Neurosciences and Signaling Group, Institute of Biomedicine (iBiMED), Department of Medical Sciences, University of Aveiro, 3810-193 Aveiro, Portugal; martinstania@ua.pt (T.S.M.);; 2 Department of Medicine, University of British Columbia, Vancouver, BC V5Z 1M9, Canada; 3 Kinexus Bioinformatics Corporation, Vancouver, BC V6P 6T3, Canada; 4 Center for Neuroscience and Cell Biology, Faculty of Pharmacy, University of Coimbra, 3004-504 Coimbra, Portugal; 5 ViraVector–Viral Vector for Gene Transfer Core Facility, University of Coimbra, 3004-504 Coimbra, Portugal; 6 Department of Psychiatry and Psychotherapy, University Medical Center Goettingen (UMG), Georg-August University, 37075 Goettingen, Germany; 7 German Center for Neurodegenerative Diseases (DZNE), 37075 Goettingen, Germany

**Keywords:** Alzheimer’s disease, biomarker, extracellular vesicles, phosphoproteome

## Abstract

Phosphorylation plays a key role in Alzheimer’s disease (AD) pathogenesis, impacting distinct processes such as amyloid-beta (Aβ) peptide production and tau phosphorylation. Impaired phosphorylation events contribute to senile plaques and neurofibrillary tangles’ formation, two major histopathological hallmarks of AD. Blood-derived extracellular particles (bdEP) can represent a disease-related source of phosphobiomarker candidates, and hence, in this pilot study, bdEP of Control and AD cases were analyzed by a targeted phosphoproteomics approach using a high-density microarray that featured at least 1145 pan-specific and 913 phosphosite-specific antibodies. This approach, innovatively applied to bdEP, allowed the identification of 150 proteins whose expression levels and/or phosphorylation patterns were significantly altered across AD cases. Gene Ontology enrichment and Reactome pathway analysis unraveled potentially relevant molecular targets and disease-associated pathways, and protein-protein interaction networks were constructed to highlight key targets. The discriminatory value of both the total proteome and the phosphoproteome was evaluated by univariate and multivariate approaches. This pilot experiment supports that bdEP are enriched in phosphotargets relevant in an AD context, holding value as peripheral biomarker candidates for disease diagnosis.

## 1. Introduction

Reversible protein phosphorylation is a key post-translational modification that regulates a multitude of physiological and pathological intracellular pathways. Alterations in protein phosphorylation patterns have been linked to several diseases, like cancer [1], diabetes [2], and neurodegenerative conditions such as Alzheimer’s disease (AD) [3]. In AD, abnormal phosphorylation events can modulate APP processing, which plays a crucial role in amyloid-beta (Aβ) peptide production, which is the main component of senile plaques [4,5]. Further, tau hyperphosphorylation leads to microtubule destabilization, consequently altered axonal transport, and also contributes to the formation of neurofibrillary tangles [6]. Indeed, many studies have focused on altered kinase and/or phosphatase expression and activities, including the impact of Aβ itself on these phosphorylation mediators [7,8]. In this disease scenario, the analysis of the AD phosphoproteome may unravel new phosphorylation-related disease pathways or novel biomarker candidates suitable for diagnostic and/or therapeutic strategies.

The current widely accepted molecular tool for AD diagnosis is based on monitoring of the gold standard biomarker triplet, Aβ1-42, total-tau, and p-tau 181 in the cerebrospinal fluid (CSF) [9,10,11]. CSF collection involves an invasive procedure only performed in hospital settings, thus limiting its wide implementation in the clinic. Hence, new biomarker candidates found in peripheral biofluids may provide an accessible diagnostic tool. Blood-derived extracellular vesicles (bdEVs) have emerged as new sources of biomarkers for AD [12,13] since these can carry disease-specific cargo and exhibit several other technical advantages, such as easy accessibility and cost-effective isolation tools. Of note, the diagnostic potential of EVs is supported by alterations in the levels of the biomarker triplet in bdEVs of AD cases [14,15,16,17] but also by proteomic analysis [18,19,20,21], lipidomic [22], and metabolic changes evaluated by Fourier Transformed Infrared Spectroscopy [23].

Protein antibody microarrays represent attractive approaches for proteome and phosphoproteome analysis since they permit monitoring both total protein expression and phosphorylation levels of hundreds of targets. Furthermore, this can be achieved using a very small sample volume in a rapid and cost-effective manner. Antibody microarrays are very sensitive and ideal for a first high-throughput analysis [24,25].

Herein, a comparative pilot analysis of blood-derived extracellular particles (bdEP), which include vesicular and non-vesicular entities, in Control and AD cases was carried out using an antibody microarray. This approach allows for the screening of hundreds of phosphosite- and pan-specific antibodies. To our knowledge, this is the first study dedicated to a large-scale bdEP phosphoproteome approach in an AD context. Novel proteins and/or phosphoproteins unveiled can constitute novel putative peripheral biomarker candidates for AD.

## 2. Results and Discussion

### 2.1. bdEP: Isolation and Characterization

**Figure 1:**
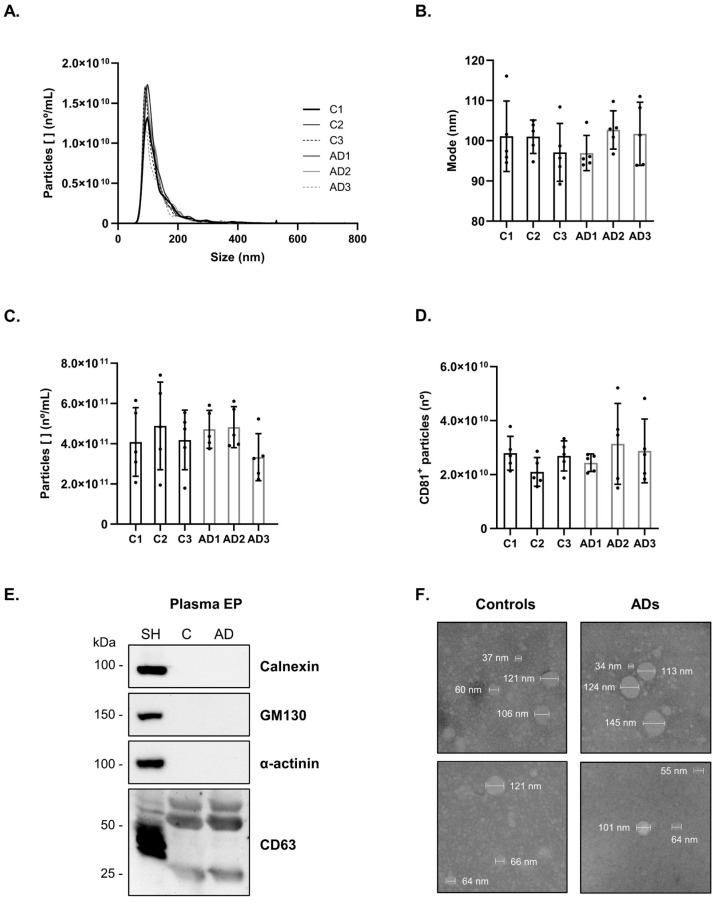
Characterization of bdEP from Controls and AD cases. bdEP batches (C1–C3 and AD1–AD3) size distribution (**A**), mode size (**B**), and particle concentration (**C**) were determined by Nanoparticle Tracking Analysis. The number of nanovesicles positive for surface marker CD81 (**D**) was determined by ELISA. Black dots represent sample measures for each pool. Western blot analysis (**E**) of the EVs marker CD63 and the negative markers Calnexin, GM130, and α-actinin. Transmission Electron Microscopy of isolated nanovesicles (**F**) from Controls or AD cases. Abbreviations: AD, Alzheimer’s disease; C, Controls; SH, SH-SY5Y cell lysates.

Blood-derived EP were isolated from the plasma of three batches of Control (C1–C3) and AD cases (AD1–AD3), each composed of five individuals. Due to the expected heterogeneity of the test subjects, this approach was adopted to focus on the most consistent differences relative to the development of AD while preserving precious samples, reducing processing costs, and still permitting a statistical analysis. The demographics and clinical data of this study groups are presented in Supplementary Table S1. The bdEP were characterized, and particle concentration was determined by Nanoparticle Tracking Analysis (NTA) and CD81 ELISA. Average size distribution curves of bdEP batches determined by NTA showed that nanovesicles isolated presented a diameter size and mode within the expected size range of 30–150 nm (Figure 1A,B). No significant differences were obtained for the size mode or particle concentration between Control and AD cases (Figure 1B,C). In addition, similar concentrations of vesicles for Control and AD cases were detected using the CD81 ELISA (Figure 1D). CD81 is a typical EVs surface marker, and its presence validates the enrichment of the preparations. Further, under these experimental conditions, Western blot analysis showed the presence of the EVs marker CD63 and the absence of Calnexin, GM130, and α-actinin (Figure 1E). It is worth mentioning that most EVs isolation methods can render preparations not completely pure, and therefore, it cannot be excluded that other non-vesicular extracellular particle contaminants, such as lipoproteins [26,27] or protein aggregates [28,29], can also be co-isolated using this precipitation-based approach. Further, Transmission Electron Microscopy (TEM) analysis showed bdEP with a round shape and within the expected sizes (Figure 1F and Supplementary Figure S1).

### 2.2. Antibody Microarray Analysis of bdEP in Controls and AD Cases

**Figure 2:**
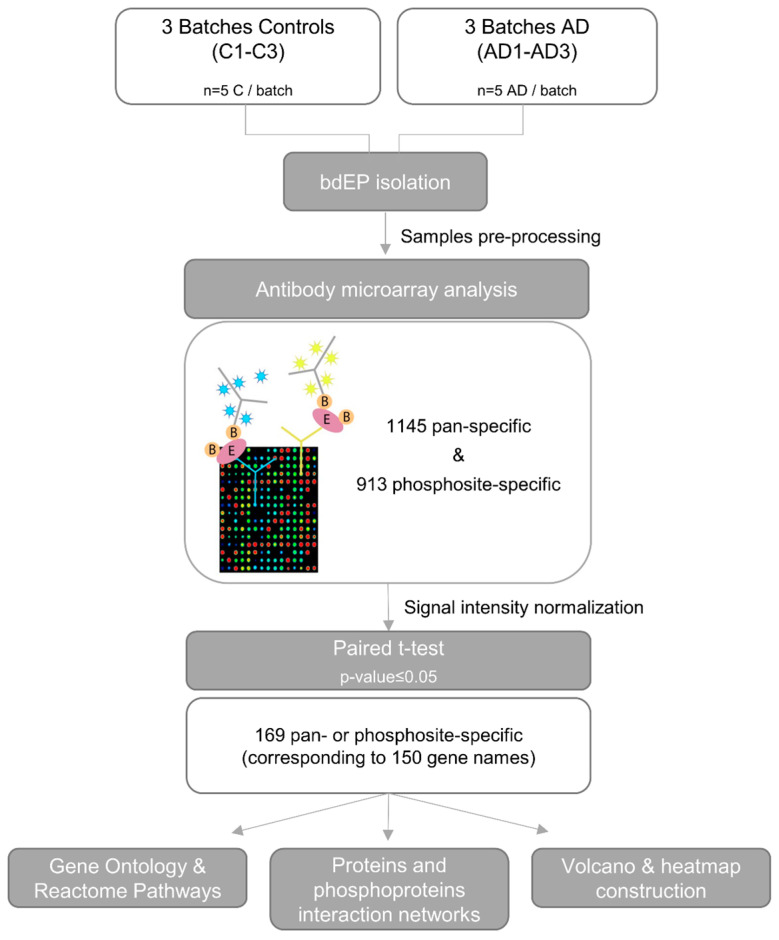
Workflow for antibody microarray analysis of bdEP isolated from Control and AD cases. Plasma samples of Control and AD cases were pooled, bdEP isolated, and further subjected to sample preparation prior to antibody microarray analysis. Globally normalized signal intensity obtained for each target was compared between the batches of Control and AD cases by paired *t*-test. Proteins that presented significantly different signal intensities were further characterized by Gene Ontology and Reactome pathway analysis. Interaction networks based on the %CFC between Controls and ADs were constructed for altered proteomes and/or phosphoproteomes. In addition, a volcano plot was constructed for the identification of most relevant proteins, and a heatmap was constructed to assess bdEP phosphoproteins discrimination of Controls and AD cases. Abbreviations: AD, Alzheimer’s disease; C, Controls; bdEP, blood-derived extracellular particles.

To detect changes in protein expression and phosphorylation, with the final goal of identifying new diagnostic biomarker candidates for AD, antibody microarray (KAM-2000) analyses were performed by Kinexus with bdEP isolated from pools of Controls and AD cases. The workflow of this pilot study is presented in Figure 2. In the antibody microarray, signal intensity for each protein was evaluated by distinct antibodies. A total of 169 pan- or phosphosite-specific antibodies, corresponding to 150 unique proteins, presented a significantly different normalized signal intensity between the bdEP of Control and AD cases (Supplementary Table S2). While some of these differences in expression or phosphorylation were small, it is noteworthy that 92% of the microarray antibodies tested did not show statistical differences, which demonstrates a high level of overall consistency between protein expression and phosphorylation in AD and Controls. Most differences in signal intensity were due to changes in pan-specific antibodies rather than in phosphosite-specific antibodies, which can be explained by the low levels of phosphorylated proteins and/or their rapid dynamics following isolation. Nonetheless, data support the idea that bdEP were able to transport phosphorylated cargo. It is important to appreciate that some of the observed changes associated with AD may have arisen from unintended cross-reactive proteins that competed for the antibodies that were printed on the KAM-2000 antibody microarray. Consequently, the key targets identified in this pilot study will need further validation by antibody-based approaches, e.g., Western blotting, which will require additional bdEP isolation from blood samples than was available for this study.

### 2.3. Gene Ontology (GO) and Reactome Pathway Analysis of the Total Proteome Altered in AD

**Figure 3:**
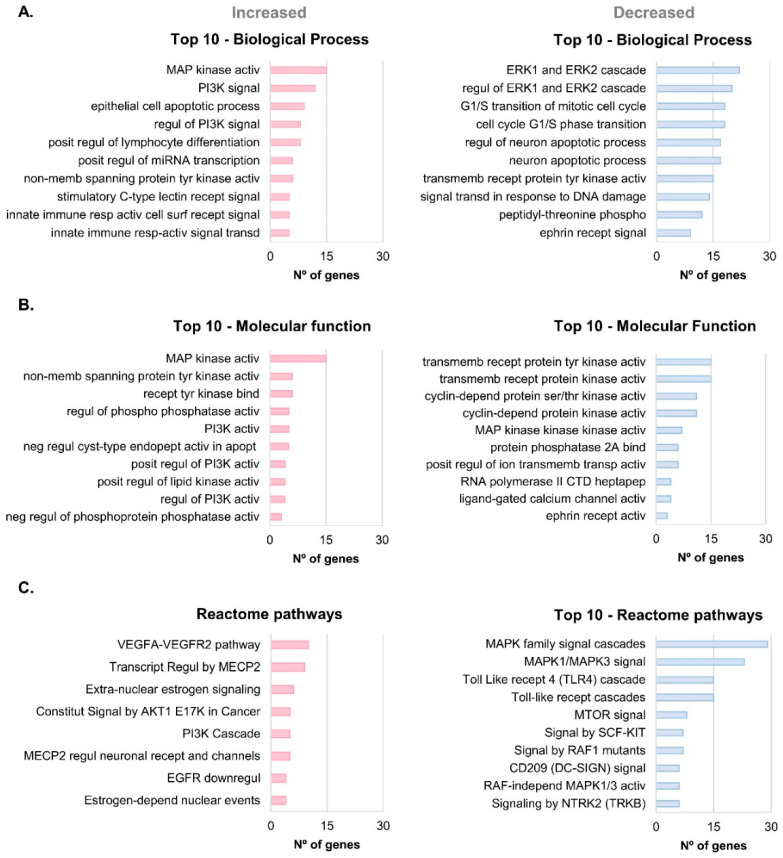
GO enrichment and Reactome pathway analysis of the significantly altered total proteome in AD. Gene Ontology enrichment analysis was performed for the significantly different total proteome between Controls and ADs, and the Top 10 biological processes (**A**) or molecular function (**B**) terms identified. Reactome pathway analysis (**C**) was also carried out. The terms with lowest *p*-value and higher number of genes associated were considered the most representative/relevant. Abbreviations: activ, activity; apopt, apoptosis; bind, binding; constitut; constitutive; cyst, cysteine; depend, dependent; downregul, downregulation; endopept, endopeptidase; heptapep, heptapeptide; independ, independent; memb, membrane; phospho, phosphorylation; posit, positive; recept, receptor; regul, regulation; resp, response; ser, serine; signal, signaling; surf, surface; thr, threonine; transcript, transcriptional regulation; transd, transduction; transmemb, transmembrane; transp; transporter; tyr, tyrosine.

Gene Ontology enrichment and Reactome pathway analyses were carried out to characterize the main processes and pathways associated with the significantly altered total proteome in AD versus Control conditions (gene names of the 150 unique corresponding proteins were used) (Figure 3).

Globally, the MAP kinase activity term was enriched for both biological processes (Figure 3A) and molecular function in bdEP of AD cases (Figure 3B). MAP kinases are protein-serine/threonine kinases involved in cell proliferation and survival, and they are divided into three families: MAPK/ERK, JNK, and p38 MAP kinases. MAP kinases were reported to be up-regulated in AD and able to promote both APP and tau hyperphosphorylation [30]. Moreover, Aβ can activate microglia, leading to oxygen reactive species production and pro-inflammatory conditions via activation of MAP signaling cascades [31].

Of note, for the decreased proteins in bdEP of ADs, the biological process ERK1 and ERK2 cascades (Figure 3A), the molecular function MAP kinase kinase activity (Figure 3B), and the MAPK family signal cascades term in the Reactome pathways (Figure 3C) were also recovered. Although this could seem contradictory, MAPK/ERK interactions are multiple and complex, involving distinct family members, some of which can act in distinct pathways and may even not interact among themselves [32]. Consistently, our group had previously shown that MAPK1/ERK2 levels were decreased in bdEP from AD cases [33].

Phosphoinositide 3-kinase (PI3K) signaling/regulation of PI3K signaling were also terms retrieved in biological processes, molecular function, and Reactome pathways analyses for the proteins increased in AD. PI3K signaling can also play a role in AD neurodegeneration by being involved in the phosphorylation of tau, for instance, by modulating GSK-3β activity [34].

The Reactome pathway with the highest number of hits, associated with increased proteins in AD, was the vascular endothelial growth factor A (VEGFA) and VEGF receptor 2 (VEGFR2) pathway (Figure 3C). VEGFA-VEGFR2 is a major pathway involved in angiogenesis. In AD, impaired angiogenic responses can ultimately contribute to cognitive decline. Aβ treatment of endothelial cells could induce a decrease in both mRNA and protein levels of VEGFR2, exerting an anti-angiogenic effect [35], and others have observed that Aβ can also interact directly with VEGFR2, blocking its activity [36]. Contrasting with our data, a decreased expression level of VEGFR2 protein was previously reported in a mixture of small (<100 nm) and larger plasma-derived EVs of AD cases [37]. Differences may arise due to the distinct EVs isolation methods that result in distinct EP subpopulations and cargo.

Molecular function terms with more hits for significantly increased or decreased proteins in AD were also related to receptor protein kinase activity, either tyr or ser/thr protein kinase activity, and phosphatase activity and binding. Data indeed indicate alterations in several kinases and phosphatases linked to AD cases.

Regarding the Reactome pathways linked to the decreased proteome, several gene names were associated with the toll-like receptor (TLR) cascade pathway. In AD, the TLR signaling cascade mediates neuroinflammation, synaptic plasticity, tau phosphorylation, and Aβ aggregation, with some controversial effects being reported. In particular, TLR4 activation can lead to both beneficial effects, promoting Aβ clearance, and detrimental effects by inducing the production of inflammatory cytokines [38,39].

Taken together, the GO and Reactome pathway analyses support that bdEP are involved in several intracellular processes and pathways and that their cargo can reflect alterations in AD pathological events.

### 2.4. AD Relevant bdEP Total Proteome Interaction Network

**Figure 4:**
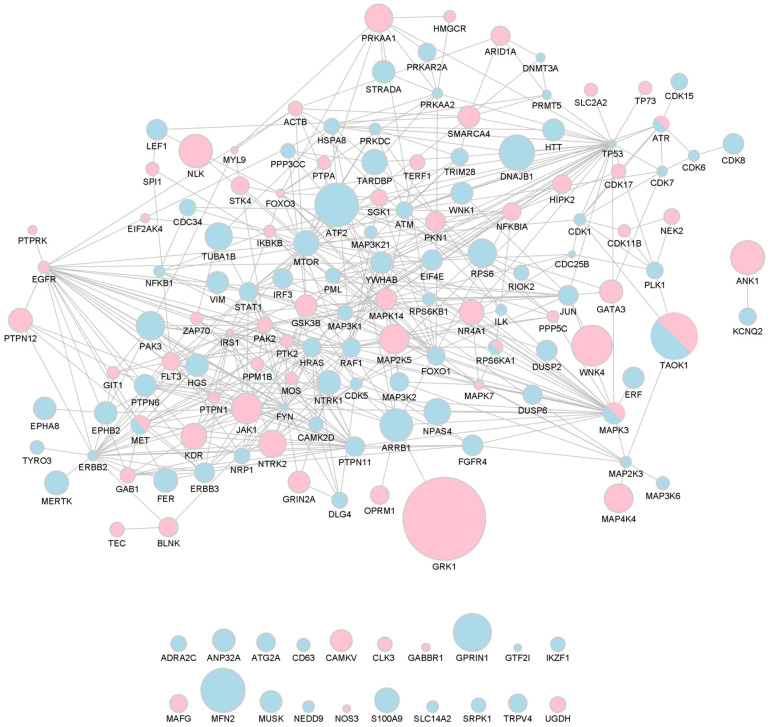
Network of bdEP proteins significantly altered in AD. Size nodes were adjusted according to %CFC. Proteins that were decreased in bdEP of AD cases are represented as blue nodes; those that were increased are represented as pink nodes; and proteins in which opposite patterns were found are represented in both colors. This network was created using STRING and imported to Cytoscape v3.9.1.

A global protein-to-protein interaction network was constructed for the 169 pan- or phosphosite-specific antibodies, corresponding to 150 unique proteins, whose signal intensity significantly changed between the bdEP of Control and AD cases (Figure 4).

The signal intensity was decreased for 85 proteins in bdEP of AD cases (represented as blue nodes), while 60 proteins were increased in bdEP of AD cases (represented as pink nodes). Five protein proteins (ATR, MAPK3 (ERK1), MET, RPS6KA1 (RSK1), and TAOK1) presented opposite variation patterns according to the antibody used (pan- or phosphosite-specific antibodies), and these were represented in both colors.

The global interaction network was constructed as percentages of change from Controls (%CFC) obtained in the microarray analysis. Since proteome patterns were evaluated using more than one antibody (either pan- or phosphosite-specific) for each target, size nodes were adjusted according to the highest %CFC (Supplementary Table S2).

GRK1, a rhodopsin kinase, was the protein that presented the highest variation, being increased in bdEP of AD cases when compared to Controls (%CFC of 114). This kinase is expressed in the retina and phosphorylates rhodopsin, which is a G-protein-coupled receptor that converts light into an electrical signal [40]. Although the role of GRK1 in AD is unclear, retinal changes have been observed in this disease. Noticeable Aβ plaques were already described in the retina, exerting neurotoxicity. A recent study also found increased Aβ1-42 levels in the retinas of AD and MCI individuals when compared to Controls, relating it to retinal atrophy and microgliosis, cognitive capacities, and the severity of Aβ and tau pathology in AD brains [41]. Additionally, the retinal proteome profiling of AD cases evidenced activation of inflammatory and neurodegenerative processes [41]. Other reports indicate that retinal changes, namely rod degeneration, could precede Aβ plaques in the brain of an AD transgenic mouse model [42]. Data support indicated that this would be an interesting target to follow, and this was subsequently addressed by complementary statistical analysis.

### 2.5. Potential of bdEP Proteome to Discrimination of AD Cases

To further assess the disease discriminatory value of the proteins that presented a significantly different signal between the bdEP of Controls and AD cases, a Principal Component Analysis (PCA) analysis was carried out. No overlap was observed between the two groups, evidencing the discriminatory power of this set of proteins (Supplementary Figure S2).

**Figure 5:**
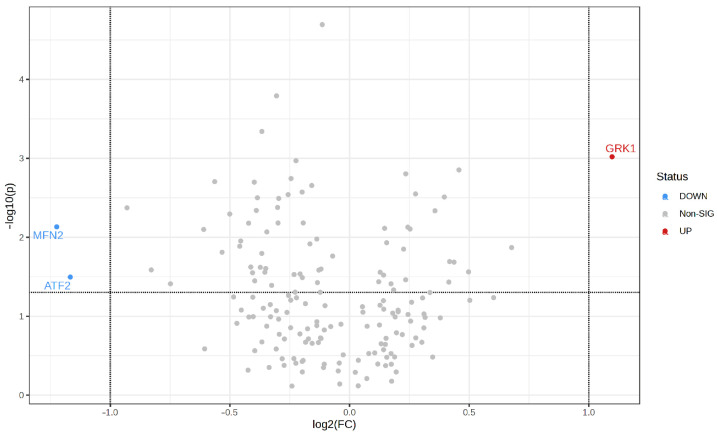
Volcano plot of total proteome in bdEP of AD cases. *Y* axis represents statistical significance (−log_10_(*p*-value)), and X axis shows fold-change alteration (log_2_FC) in normalized signal intensity of proteins. Red dot indicates a statistically significantly increased protein, and blue dots depict decreased proteins between bdEP of Controls and AD cases. Horizontal dashed line indicates a *p*-value threshold of *p* ≤ 0.05, and vertical line represents the AD/Controls fold-change threshold of >2 (log_2_FC > 1 or <−1). Volcano plot was constructed on MetaboAnalyst 5.0. Abbreviations: FC, fold-change; *p*, *p*-value; sig, significant.

Among the proteins with significantly different signal abundance in AD cases, those that most changed were GRK1, as already mentioned, but also MFN2 and ATF2 (volcano plot, Figure 5). These three proteins were detected with pan-specific antibodies and presented the highest signal abundance alterations between AD cases/Controls (log_2_ fold change > 1 or <−1), more than 2-fold, and with statistical significance (*p* ≤ 0.05).

MFN2 protein expression levels were highly decreased in bdEP of AD cases in comparison to Controls (%CFC of −57%). MFN2, or mitofusin-2, is a mitochondrial membrane protein that is involved in the fusion and maintenance of the mitochondrial network. In accordance with our data, others observed decreased mRNA or MFN2 protein levels in the brains of AD patients or AD transgenic mice [43,44]. Further, it was shown that MFN2 downregulation could impair γ-secretase activity and decrease Aβ production by increasing endoplasmic reticulum and mitochondrial coupling [45,46], although through a poorly understood mechanism.

ATF2 is a cAMP-dependent transcription factor whose levels were decreased in bdEP of AD cases when compared to Controls (%CFC of −56%). This factor is mainly expressed in the brain and can regulate the transcription of genes associated with cell homeostasis, DNA damage, and death-survival mechanisms [47]. Of note, ATF2 can be activated by the MAPK pathway, which is also reported to be altered when MAPK signaling is induced by Aβ, promoting astrocyte inflammatory responses and neuronal damage [47,48]. ATF2 changes in AD need to be further explored since this transcription factor was found to be downregulated in the hippocampus [49,50] but up-regulated in the cerebral cortex of AD human brains [51,52].

### 2.6. AD bdEP Phosphoproteins Network

**Figure 6:**
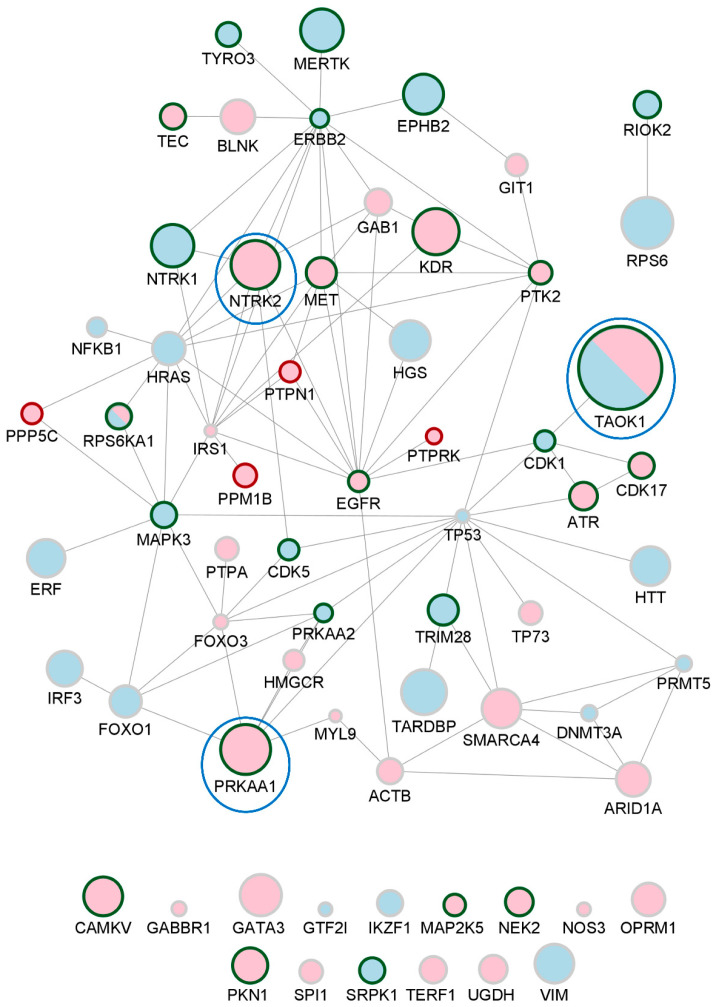
Network of bdEP phosphoproteins significantly altered in AD. Size nodes were adjusted according to %CFC, and top 3 nodes with higher %CFC are surrounded by a blue circle. Phosphoproteins that were increased in bdEP of AD cases are represented as pink nodes; decreased phosphoproteins are represented as blue nodes. For TAOK1 and RPS6KA1, phosphorylated residues were found to be increased or decreased, and, thus, these phosphoproteins are represented in both colors. Kinases and phosphatases are highlighted by node border colors of green or red, respectively. These phosphoprotein networks were created using STRING and imported to Cytoscape v3.9.1.

Considering the relevance of protein phosphorylation in AD pathology, an interaction network specific for the phosphoproteins that significantly changed in bdEP of AD cases was constructed (Figure 6). From the 150 significantly different proteins, 65 unique UniProt IDs, corresponding only to phosphosite-specific antibody signal intensity, were used as an input for the network construction in the STRING database. The signal intensity was decreased for 27 phosphoproteins in bdEP of AD cases (represented as blue nodes), while 36 phosphoproteins were increased in bdEP of AD cases (represented as pink nodes). For RPS6KA1 and TAOK1 (as described above), opposite phosphorylation patterns were observed for different residues (phosphoproteins are represented in both colors). In addition, 26 kinases and 4 phosphatases with significantly altered phosphorylation patterns were highlighted (green and red contours, respectively).

In the specific phosphoproteins interaction network, several kinases with relevance for tau and APP metabolism could be identified, including TAOK1, PRKAA1, and NTRK2, which exhibited higher %CFC in AD.

The TAOK1 gene encodes for the protein-serine/threonine kinase TAO1, a tau kinase that exhibits opposite phosphorylation patterns depending on the residue. The phosphorylation levels at S181 were increased (%CFC of 60) while decreased at Y309 (%CFC of −22) in bdEP of AD cases. Active TAO1 kinase, phosphorylated at S181, was found in AD brain sections at distinct tau pathology stages, co-localizing with NFTs [53]. In addition, phosphorylation of TAO1 kinase at S181 is higher in AD brain sections than in Controls [53]. The relevance of TAO1 kinase phosphorylation at Y309 still remains to be elucidated.

The PRKAA1 gene encodes for the catalytic subunit of AMP-activated protein kinase (AMPK), a heterotrimeric protein-serine/threonine kinase that plays a key role in AD metabolism [54]. AMPK is likewise a tau kinase, found to be dysregulated in AD brains, co-localizing with tau tangles [55]. Additionally, studies indicate isoform-specific roles in AD [56], with increased levels of AMPKα1 and reduced levels of AMPKα2 found in the hippocampus of postmortem AD brains when compared to Controls [56]. Herein, AMPK1α phosphorylation at T183/S184 increased in bdEP of AD cases when compared to Controls (%CFC of 33). AMPKα1 is activated by phosphorylation in T183 [57], and the observations reported in bdEP support its activation.

NTRK2, also known as TRKB (tyrosine kinase receptor B), encodes for the receptor for brain-derived neurotrophic factor (BDNF), involved in synaptic plasticity and neuronal development. This gene, expressed in the central and peripheral nervous systems, was proposed as a risk gene for AD [58]. BDNF can bind to the TrkB receptor, leading to its dimerization and autophosphorylation. Interestingly, it was also shown that TrkB could bind and phosphorylate APP at Y687, decreasing APP amyloidogenic cleavage and Aβ production [59]. In AD, both BDNF and TrkB were found to be decreased in transgenic mice’s brains, contributing to impaired signaling and exacerbated memory deficits without interfering with amyloidosis [60]. In our study, TrkB phosphorylation at Y706/Y707 was increased in bdEP of AD cases (%CFC of 32) when compared to Controls. Phosphorylation of TrkB at these residues may be induced by BDNF, and this is essential for TrkB activation [61]. Nonetheless, phosphorylation of additional tyrosine residues is required for the activation of downstream signaling pathways.

Overall, the phosphoproteins network presented in Figure 6 highlighted novel phosphorylation patterns altered in bdEP of AD cases, which may reflect alterations in disease-related signaling pathways and constitute putative phosphotargets.

### 2.7. bdEP Phosphoproteins in the Discrimination of AD Cases

**Figure 7:**
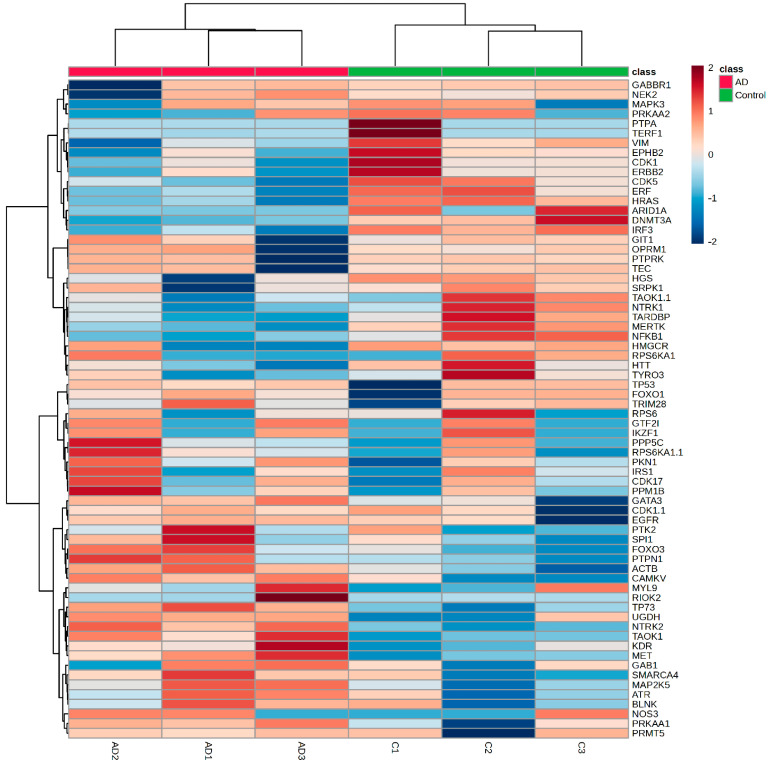
Heatmap for phosphoproteins that significantly changed between the bdEP of Controls and AD cases. Heatmap was constructed on MetaboAnalyst. The bars on the top of heatmap show each batch of Controls (C1–C3) or AD cases (AD1–AD3). Red represents higher signal intensity, and blue represents lower signal intensity.

To address the discriminatory value of the phosphoproteins identified in bdEP, a heatmap was constructed (Supplementary Table S3). This unsupervised hierarchical analysis split up the samples into two sets: Controls and AD cases (Figure 7). PCA analysis also supported that this set of phosphoproteins in bdEP exhibits disease-discriminatory value (Supplementary Figure S3). Taken together, the data support the potential of these comparative pilot phosphoproteomic analyses in distinguishing Controls from ADs and identifying targets for subsequent validation.

## 3. Materials and Methods

### 3.1. Study Group Characterization

Plasma samples of Control and AD cases were obtained from the biobank of the Universitätsmedizin Göttingen. The pseudonymized collection of biological samples and clinical data and their use in biomarker studies were approved by the ethics committee of the University Medical Center Goettingen (approval 9/2/16). All subjects or their legal representatives gave their informed consent prior to inclusion. Biomaterial sampling and data collection were conducted according to the revised Declaration of Helsinki and good clinical practice guidelines.

Plasma was collected in EDTA Monovettes (S-Monovette 9 mL K3E; Sarstedt, Nümbrecht, Germany), centrifuged at 2000× *g* for 10 min, at room temperature (RT), to obtain EDTA blood plasma. Samples were aliquoted and stored at −80 °C. The Controls group included 15 age-matched individuals with a mean age of 65.60 ± 9.36 years, and the AD group comprised 15 individuals with a mean age of 67.00 ± 9.82 years (*p*-value = 0.69). AD diagnosis followed the 2011 McKhann criteria and included cognitive testing (Mini-Mental State Examination and Clock-Drawing Test), CSF neurochemical biomarker triplet assessment, and/or PET scan imaging.

### 3.2. Blood-Derived EP: Isolation and Characterization

Three batches were prepared, each of which involved pooling plasma samples from five age-matched Controls (C1–C3) or AD cases (AD1–AD3). The demographics and clinical data of the individuals included in this study are available in Supplementary Table S1. Each batch of 125 μL of plasma (25 µL of each individual) was then used for bdEP isolation using the precipitation-based method ExoQuick. In brief, plasma batches were centrifuged at 3000× *g* for 15 min at RT. Further, 100 μL of supernatant were mixed with 25 μL of the precipitation reagent, incubated for 30 min at 4 °C, and centrifuged at 1500× *g* for 30 min at 4 °C. The obtained pellet was then resuspended in 200 μL of Kinexus Lysis Buffer for antibody microarray analysis.

For bdEP characterization by ELISA and Western blot analysis, EP-enriched pellets were resuspended in PBS or RIPA buffer, respectively. The protein concentration of bdEP was determined by the BCA protein assay. The CD81 detection was achieved using the ExoELISA-ULTRA Complete Kit and 25 μg of total protein in the batch sample. Other EVs markers were also assessed by Western blot analysis, and 50 µg of bdEP were loaded on a 5–20% SDS-PAGE followed by overnight protein transfer to nitrocellulose membranes. These were blocked with 5% non-fat dry milk during 4 h and incubated overnight at 4 °C with anti-CD63 (1:500) (sc-5275; Santa Cruz Biotechnology, Dallas, TX, USA), anti-α-actinin (1:500) (612576; BD Transduction Laboratories, Franklin Lakes, NJ, USA), anti-GM130 (1:500) (610822; BD Transduction Laboratories, Franklin Lakes, NJ, USA), and anti-Calnexin (1:200) (ADI-SPA-860-J, Enzo, Farmingdale, NY, USA). After, membranes were incubated with anti-mouse IgG, HRP-linked antibody (1:2000 for anti-CD63 and 1:10,000 for anti-α-actinin and anti-GM130) (7076S; Cell Signaling Technology, Danvers, MA, USA) or anti-rabbit IgG, HRP-linked antibody (1:2000 for anti-Calnexin) (7074S; Cell Signaling Technology, Danvers, MA, USA) for 2 h at RT. Protein bands were detected using ECL Select reagent (GE Healthcare Life Science, Milwaukee, WI, USA), and the chemiluminescence images were acquired with the ChemiDoc gel imaging system (Bio-Rad, Hercules, CA, USA).

For bdEP characterization by TEM and NTA, the final pellet was resuspended in PBS. TEM and NTA analyses followed the procedures previously described by the group [23]. For TEM analysis, a negative staining with phosphotungstic acid solution was performed, and the images were captured using a STEM Hitachi HD 2700 microscope at 100 kV. For NTA, bdEP were diluted 1:1000 in PBS and analyzed using the NanoSight NS300 (Malvern Instruments) and the NTA software version 3.2. The particle concentrations obtained were multiplied by the dilution factor before statistical analysis. The data distribution of EP mode size and particle concentration were evaluated by the Shapiro–Wilk test, and EP size or concentration between batches were compared using the non-parametric Kruskal–Wallis test.

### 3.3. Blood-Derived EP Preparation for Antibody Microarray

Prior to the Kinexus KAM-2000 antibody microarray analysis, bdEP pellets of Control and AD cases (batches C1–C3 and AD1–AD3) were resuspended in Kinexus Lysis Buffer with phosphatase inhibitors plus protease inhibitor cocktail (cOmplete, Mini Protease Inhibitor Cocktail from Roche), dithiothreitol, and Tris(2-carboxyethyl)phosphine hydrochloride) to reduce disulfide bonds. Since the antibody microarray uses non-denatured proteins, it is necessary to reduce protein complexes as much as possible to prevent false positives. Hence, a chemical cleavage step was performed after bdEP pellet lysis to cleave the proteins and dissociate the complexes, reducing false positives and improving the phosphorylation state stability of target proteins by inhibiting the activity of endogenous kinases, phosphatases, and proteases. In addition, this step ensures that the strength of the signals detected does not depend on the size of the target proteins. In brief, after lysis buffer addition, the pH was adjusted to 9, and samples were sonicated 4× to complete membrane lysis and shear of nuclear DNA. Further, 2-nitro-5-thiocyanatobenzoic acid at a final concentration of 6 mM was added, pH adjusted again to 9, and homogenates were incubated at 37 °C for 15 min in a water bath. Afterwards, bdEP lysate homogenates were cleared by centrifuging at 90,000× *g* for 30 min at RT. The supernatants were then transferred to new tubes, and the pH was adjusted to 7. Protein concentrations were determined by Bradford assay, and a total of 200 μg of bdEP lysates were sent to Kinexus Bioinformatics Corporation for Kinex KAM-2000 antibody microarray analysis. Lysates of the chemically cleaved bdEP were labeled with biotin and applied to the microarray, which contained at least 1145 pan-specific capture antibodies and 913 phosphosite-specific antibodies that were immobilized on the array slides. After, microarray was probed with a fluorescent dye-labeled anti-biotin antibody, and images of each array were captured using a Perkin-Elmer ScanArray Reader laser array scanner. Duplicate measurements of fluorescence signals were performed for each captured target protein with an antibody. The simultaneous use of these pan- and phosphosite-specific types of antibodies for each target permits the identification of specific changes in phosphorylation stoichiometry by comparing them with total protein expression levels. For each antibody microarray, output included globally normalized signal intensity and a standard deviation for duplicates. Percentages of change from Controls (%CFC) were also calculated between signals captured for each target [((Globally normalized signal in AD/Globally normalized signal in Control) × 100) − 100]. Negative %CFC values indicate the percentage reduction in signal intensity in AD cases in relation to the Control, while positive %CFC values indicate an increase in signal intensity for AD cases. A %CFC of 100% indicates a 2-fold increase in signal intensity for AD cases, and a negative %CFC of −50% indicates that signal intensity has reduced by half in ADs when compared to Controls. The typical median percent error in the duplicate measurements performed in this study was 5%, and the average of the duplicate measurements was used for comparisons.

Subsequent analysis focused only on proteins that presented a significantly different globally normalized signal intensity for protein expression levels and/or phosphorylation patterns.

### 3.4. Gene Ontology and Reactome Pathway Analysis

GO enrichment for the molecular function and biological process, and Reactome pathway analyses were carried out for the significantly altered total proteome (corresponding gene names were used), comparing Control and AD cases (*p* ≤ 0.05). These analyses were performed using Cytoscape (version 3.9.1) and the ClueGo plug-in (version 2.5.9) [62,63] on 17 July 2023. UniProt IDs of proteins that increased or decreased in relation to AD cases were uploaded in separated clusters to identify specific associated terms. The statistical test “Enrichment/Depletion (Two-sided hypergeometric test)” and p-value correction “Bonferroni step down” were selected. Only GO and Reactome terms with a *p*-value ≤ 0.05 were considered, and for both clusters, the GO Tree Interval used ranged from 5 to 10 levels. For the analysis of the significantly altered total proteome in AD, the top 10 GO processes or Reactome pathway terms with a lower p-value and a higher number of hits associated were considered the most representative.

### 3.5. Protein Interaction Network Construction

Total proteome or phosphoproteome interaction networks were retrieved from the STRING online database (https://string-db.org/, accessed on 17 July 2023). UniProt IDs of significantly altered proteins were added as input into STRING, and active interaction sources selected were “experiments” and “databases”. The resulting protein networks were then imported into Cytoscape. Node size was adjusted according to %CFC to highlight the nodes with the highest percentage of change between AD cases and Controls.

### 3.6. Microarray Statistical Analysis

For microarray analysis, normalized signal abundance captured with phosphosite- and pan-specific antibodies was compared between bdEP of Control and AD cases by paired *t*-test (*p* ≤ 0.05). Each microarray was performed with a batch of Controls (C1–C3) and a batch of AD cases (AD1–AD3). This analysis was performed using GraphPad 9 software.

Univariate statistical analyses comprising volcano plots and heatmaps and multivariate statistical analyses, including PCA, were carried out using MetaboAnalyst 5.0 (https://genap.metaboanalyst.ca/MetaboAnalyst/, accessed on 20 July 2023) [64].

To identify the proteins with the higher normalized signal abundance changes, a volcano plot was constructed. The fold change threshold was set to 2, and statistical significance was set to *p* ≤ 0.05. For each protein, the *p*-value of the *t*-test was plotted against the base 2 logarithm of the fold change ratio of signal abundance obtained in AD cases relative to Controls.

A heatmap was constructed to assess the clustering of samples from each group according to globally normalized signal intensity. The Eucledian distances were calculated, and the Ward clustering method was used. The color scale represents the normalized signal intensity.

Multivariate Analysis was also performed to assess the discrimination between Controls and AD cases. PCA was employed to address Control and AD group segregation on the 2D score plot.

## 4. Conclusions

In sum, the pilot study herein presented reveals a set of bdEP proteins whose altered total expression levels or phosphorylation status can represent putative targets for AD diagnosis. Although their enrollment in disease pathogenesis has been proven for most of the highlighted putative targets, very scarce studies have evaluated their levels in peripheral biofluids (Table 1). This might relate to their low levels in peripheral biofluids and to the unavailability of ELISA kits, especially for the phosphoproteins. Nevertheless, the microarray approach presented allowed us to measure these targets, linking them to AD. The association of these proteins with the disease supports their potential as putative biomarkers. Future studies should address the levels of these targets in bdEP using high-sensitivity methodologies and large cohorts, including other types of dementia, for a differential diagnosis evaluation.

The bdEP comparative phosphoproteome linked to AD was revealed for the first time, supporting that bdEP cargo is indeed involved in key disease pathogenic pathways. Several putative biomarker candidates were identified in bdEP, including kinases and phosphatases, emphasizing the importance of these extracellular particles as a peripheral, non-invasive diagnostic tool for AD.

## Figures and Tables

**Table 1:** Proteins altered in bdEP may constitute putative biomarker candidates for AD. Protein signal abundance detected in the microarray analysis of bdEP isolated from Controls and AD cases. Their role and alteration pattern in AD brain are summarized.

Gene Name	UniProt ID	Antibody P-Site	C Mean	AD Mean	%CFC	*p*-Value	Relevance in AD	Levels in AD Brain	Ref
MFN2	O95140	Pan	2410	1032	−57	0.009	MFN2 down-regulation impairs γ-secretase activity and decreases Aβ production; Decreased mRNA and protein levels in the brains of AD patients and AD transgenic mice	↓ mRNA and protein in humans or mice	[43,44,45,46]
ATF2	P15336	Pan	317	141	−56	0.020	MAPK signaling, induced by Aβ, activates ATF2, resulting in an inflammatory response by astrocytes	↓/↑ In humans	[47,48,49,50,51,52]
NTRK2	Q16620	Y706 + Y707	1708	2247	32	0.039	Risk gene in AD: Binds and phosphorylates APP at Y687, retaining APP in TGN, and decreasing Aβ production	↓ pan in humans and mice	[58,59,60]
PRKAA1	Q13131	T183 + S184	4926	6566	33	0.039	Tau kinase, co-localized with neurofibrillary tangles	↑ pan in human	[55,56]
TAOK1 *	Q7L7 × 3	S181	262	419	60	0.023	Tau kinase, active and co-localized with neurofibrillary tangles	↑ active (S181) human	[53]
GRK1	Q15835	Pan	288	615	114	0.017	Unknown	-	-

* For TAOK1, distinct phosphorylated residues were found to be either increased or decreased. Abbreviations: AD, Alzheimer’s disease; C, Controls; CFC, Percentage of change from Controls; TGN, Trans-Gogi network; ↓ decreased; ↑ increased.

## Data Availability

Data are contained within the article and supplementary materials.

## Supplementary Materials

The following supporting information can be downloaded at: https://www.mdpi.com/article/10.3390/ijms25031584/s1.
