# Supplementary material for: Phosphoproteome Microarray Analysis of Extracellular Particles as a Tool to Explore Novel Biomarker Candidates for Alzheimer’s Disease
Source: Int J Mol Sci. 2024 Jan 27;25(3):1584. doi: 10.3390/ijms25031584 (PMC10855802; doi:10.3390/ijms25031584)
Supplement: Supplementary file 1 [file ijms-25-01584-s001.zip › ijms-2776393-supplementary.pdf]

Manuscript: Phosphoproteome Microarray Analysis of Extracellular Particles as a Tool to Explore Novel Biomarker Candidates for Alzheimer's Disease

Supplementary Materials

Supplementary Figure S1

Control **AD**

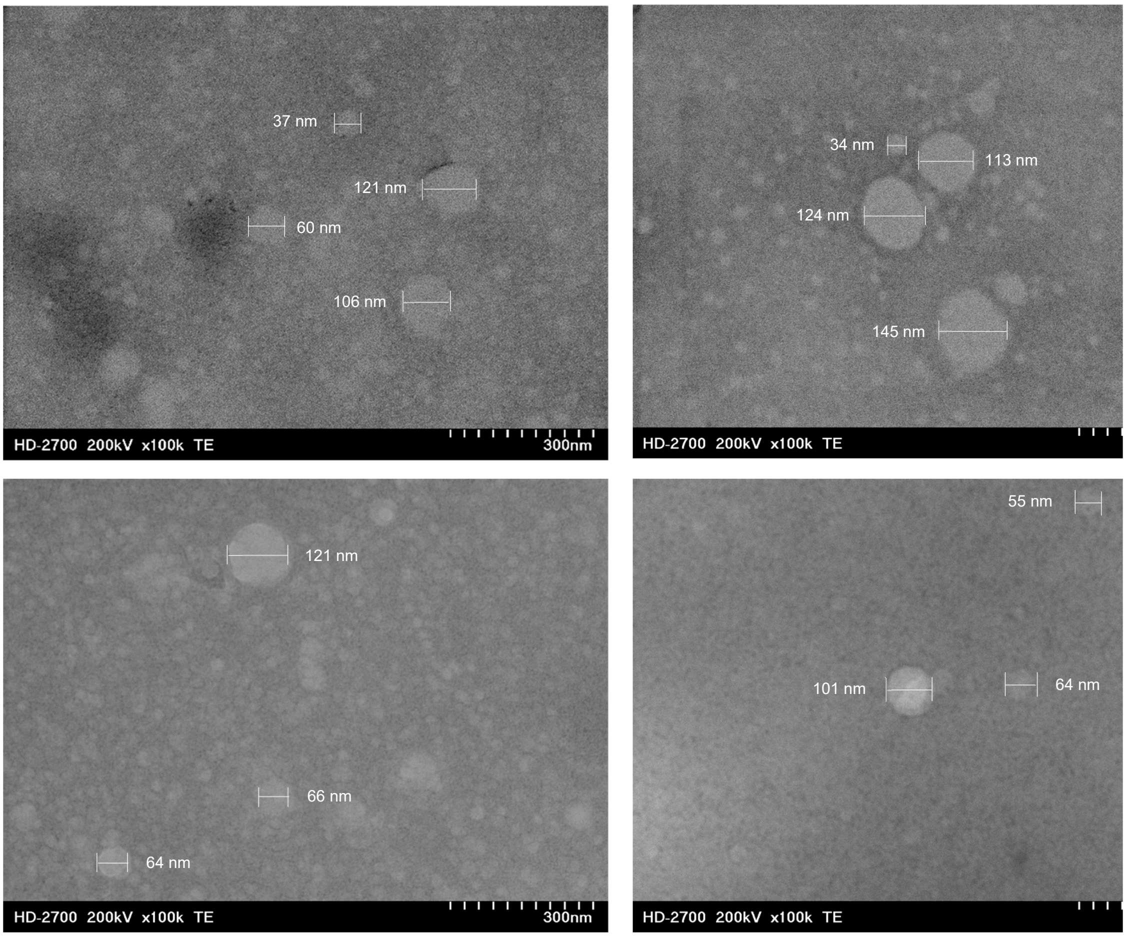

(A) **(B)**

Supplementary Figure S1. TEM image frames for Controls and AD cases. Large size entire TEM frames depicted in the manuscript Figure 1 for Controls (**A**) or AD cases (**B**).

Supplementary Figure S2

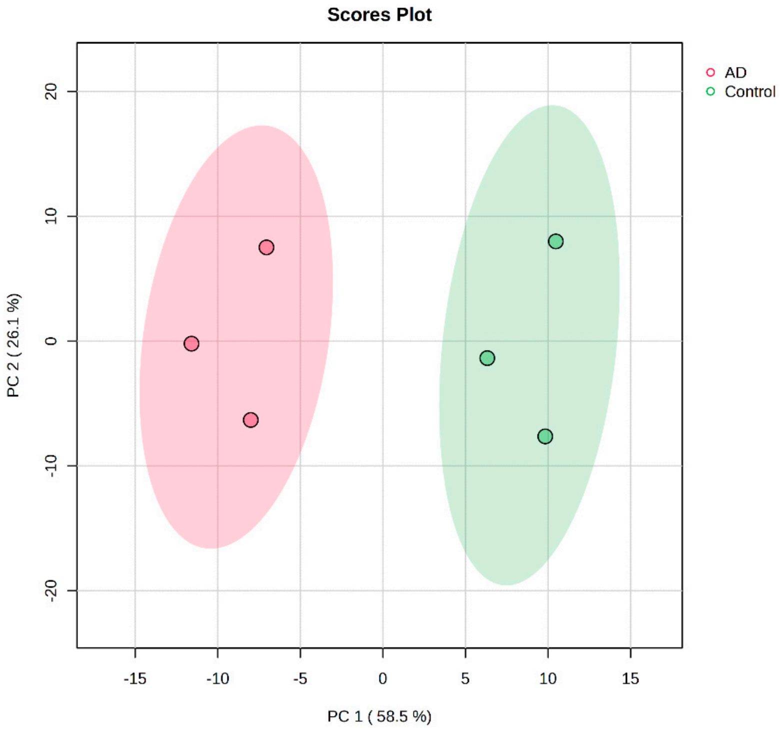

Supplementary Figure S2. PCA of bdEPs proteome significantly different in AD cases. A PCA score plot was performed to assess the discrimination between Controls (C1-C3 batches) and AD cases (AD1-AD3 batches). In score plot, the green and pink areas represent the 95% confidence region.

Supplementary Figure S3

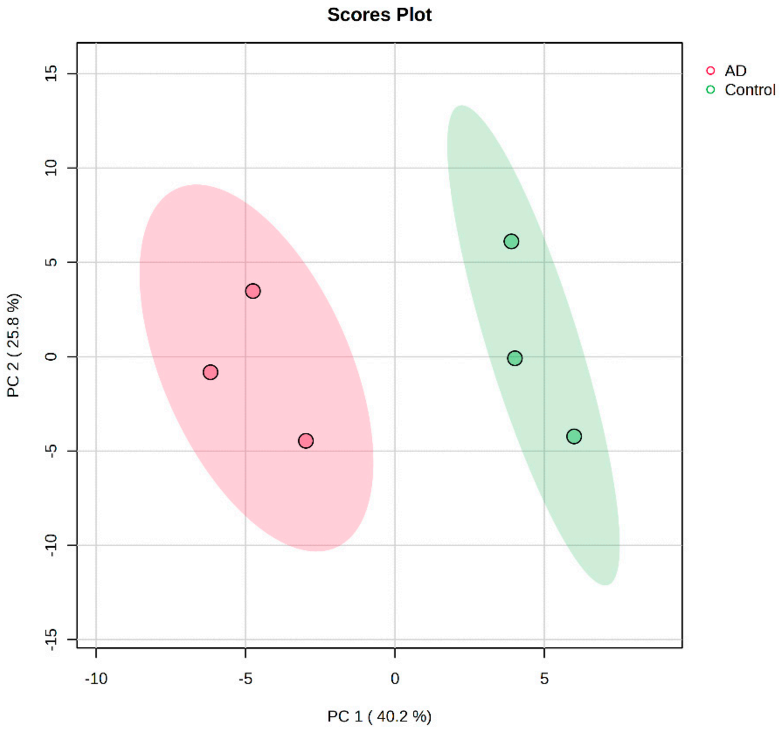

Supplementary Figure S3. PCA of bdEPs phosphoproteins that significantly changed in AD cases. A PCA score plot was performed to assess the discrimination between Controls (C1-C3 batches) and AD cases (AD1-AD3 batches). In score plot, the green and pink areas represent the 95% confidence region.

Supplementary Tables

Supplementary Table S1

Supplementary Table S1. Demographics and clinical data of Controls and AD cases. Data distribution was assessed by Shapiro–Wilk and age; cognitive tests and CSF biomarker concentrations were compared using the non-parametric Kruskal–Wallis test.

	Controls			AD cases			Controls vs AD cases
	Batch C1	Batch C2	Batch C3	Batch AD1	Batch AD2	Batch AD3	P-value
**Age** (mean±SD)	64.40±10.33	66.00±5.57	66.40±12.88	65.60±10.48	67.80±11.54	67.60±9.53	0.994
**CDT** (points) (mean±SD)	1.00±0.00	1.67±1.16	1.80±0.84	2.40±1.34	3.25±0.50	3.40±1.14	0.040*
**MMSE scores** (mean±SD)	23.75±3.30	29.00±1.73	28.5±1.73	22.40±4.62	21.60±4.62	24.00±2.92	0.031**
**CSF Aβ1-42** (ng/mL) (mean±SD)	1198.0±136.8	1388±638.2	1437.0±593.0	602.8±100.7	606.8±134.5	618.8±135.9	0.002
**CSF Aβ1-40** (ng/mL) (mean±SD)	8879±2395	10216±2387	11219±3559	13034±2610	12676±5932	13183±5481	0.354
**CSF Aβ1-42/1-40** (mean±SD)	1.40±0.24	1.35±0.58	1.31±0.34	0.47±0.11	0.54±0.33	0.50±0.11	0.001
**CSF P-tau 181** (pg/mL) (mean±SD)	42.40±7.50	42.60±14.64	52.00±16.93	100.60±39.83	100.6±34.63	74.80±25.12	0.003
**CSF T-tau** (pg/mL) (mean±SD)	221.20±44.03	242.80±90.26	193.00±82.40	985.00±598.30	838.80±359.00	472.60±170.40	<0.001

Abbreviations: AD, Alzheimer's disease cases; C, Controls; CDT, Clock-Drawing Test; CSF, Cerebrospinal fluid; MMSE, Mini-Mental State Examination; SD, Standard deviation. * Data available for n=11 Controls and n=15 ADs. ** Data available for n=11 Controls and n=14 ADs.

Supplementary Table S2. Total proteome significantly changed between Controls and AD cases. Proteins detected with pan- and/or phosphosite-specific antibodies were included. Average signal intensity and standard deviation were calculated for the 3 batches of Controls (C1-C3) or the 3 batches of AD cases (AD1-AD3). A total of 150 proteins were found to be significantly altered in AD. The changes in signal intensity between Controls and AD cases are presented as the percentage change from Controls (%CFC). The protein signal decrease in AD cases is represented in blue, and the signal increase is represented in light pink. Signal intensities obtained for each target between 3 batches of age-matched Controls and 3 batches of AD cases were compared using a paired t-test.

					Controls			AD				
**Target Name**	**Antibody P-Site**	**Cat No.**	**UniProt ID**	**Gene name**	**Mean**	**S.D.**	**S.D. %**	**Mean**	**S.D.**	**S.D. %**	**%CFC**	**p-Value**
MFN2 (Marf; CPRP1)	Pan	NN290-1	O95140	MFN2	2410	315	13	1032	226	22	-57	0.009
ATF2 (CRE-BP1)	Pan	9222	P15336	ATF2	317	65	20	141	42	30	-56	0.020
GRIN1 (NMDAR1; GPRIN1)	Pan	NN264-1	Q7Z2K8	GPRIN1	1467	88	6	770	143	19	-48	0.020
HSP40 (DNAJB1; DNAJ1; HDJ1; HSPF1)	Pan	NN057-2	P25685	DNAJB1	1660	242	15	935	172	18	-44	0.005
Arrestin b	Pan	610551	P49407	ARRB1	2216	403	18	1318	116	9	-40	0.049
PAK3 (PAKb)	Pan	sc-1871	O75914	PAK3	478	47	10	313	7	2	-34	0.048
RPS6	S235+ S236	PN685	P62753	RPS6	3221	994	31	2116	650	31	-34	0.049
Tubulin-alpha (TUBA1B)	Pan	NN380-1	P68363	TUBA1B	248	13	5	168	8	5	-32	0.009
NPAS4 (BHLHE79; NXF; PASD10)	Pan	NN299-1	Q8IUM7	NPAS4	2046	114	6	1415	189	13	-31	0.008
mTOR (FRAP)	Pan	NK116-4	P42345	MTOR	15776	353	2	11148	1119	10	-29	0.024
TARDBP	S409+ S410	PN674	Q13148	TARDBP	1994	259	13	1424	160	11	-29	0.030
S100A9	Pan	NN459-3	P06702	S100A9	3462	573	17	2497	402	16	-28	0.019
Fer (TYK3)	Pan	AP7704b	P16591	FER	159	13	8	116	5	4	-27	0.039
MERTK (MER)	Y753	PK704	Q12866	MERTK	1919	141	7	1400	84	6	-27	0.028
TrkA (NGFR; NTRK1)	T496	sc-8058	P04629	NTRK1	35	5	14	26	3	12	-27	0.024
HGS (Hrs)	Y216	PN519	O14964	HGS	3876	260	7	2893	66	2	-25	0.049
EphB2	Y780	PK610	P29323	EPHB2	3076	403	13	2298	330	14	-25	0.015
STRAD (STLK5)	Pan	sc-515635	Q7RTN6	STRADA	85	30	36	63	25	39	-25	0.037
I1PP2A (ANP32A/B; PHAPI)	Pan	B13007	P39687	ANP32A	3182	232	7	2390	213	9	-25	0.026

EphA8	Pan	AP7613a	P29322	EPHA8	1102	110	10	831	24	3	-25	0.050
VIM (Vimentin)	S34	KAM-CC246	P08670	VIM	388	30	8	294	41	14	-24	0.019
Huntingtin (HTT)	S421	PN829	P42858	HTT	3191	378	12	2413	355	15	-24	0.009
MuSK	Pan	sc-6010	O15146	MUSK	1914	73	4	1452	54	4	-24	0.021
WNK1	Pan+ S382	PK855	Q9H4A3	WNK1	583	60	10	443	20	5	-24	0.044
PTPN6 (PTP1C; SH-PTP1)	Pan	sc-7289	P29350	PTPN6	109	19	17	83	22	27	-24	0.029
14-3-3 (KCIP-1)	Pan	NN441-2	P31946	YWHAB	3359	90	3	2564	174	7	-24	0.006
ERK1 (MAPK3; ERT2)	Pan	NK055-1	P27361	MAPK3	24963	1145	5	19112	623	3	-23	0.031
ERF	T526	PN584	P50548	ERF	7199	526	7	5558	391	7	-23	0.011
DUSP2 (PAC1)	Pan	NP008-4	Q05923	DUSP2	11746	411	3	9107	835	9	-22	0.048
eIF4E	Pan	610270	P06730	EIF4E	3389	87	3	2628	53	2	-22	0.011
TAO1 (TAOK1)	Y309	PK827	Q7L7X3	TAOK1	2974	522	18	2307	363	16	-22	0.031
FGFR4	Pan	NK239-1	P22455	FGFR4	1899	166	9	1479	191	13	-22	0.012
CDK8	Pan	sc-1521	P49336	CDK8	1331	48	4	1041	111	11	-22	0.035
LEF1	Pan+ T155	PN616	Q9UJU2	LEF1	1602	116	7	1256	77	6	-22	0.035
PTPN6 (PTP1C; SH-PTP1)	Pan	P17320 610126	P29350	PTPN6	606	86	14	476	46	10	-21	0.045
IRF3	T135	PN610	Q14653	IRF3	3146	84	3	2476	178	7	-21	0.043
Jun (c-Jun)	Pan	sc-74543	P05412	JUN	34	8	25	27	8	32	-21	0.021
ErbB3 (HER3)	Pan	NK231-2	P21860	ERBB3	6350	445	7	5046	610	12	-21	0.012
PTPN11 (PTP1D; PTP2C; SHP2; SHPTP2; Syp)	Pan	P54420 610622	Q06124	PTPN11	995	114	12	792	73	9	-20	0.039
DUSP6	Pan	NP040-3	Q16828	DUSP6	5591	454	8	4461	284	6	-20	0.012
Raf1 (c-Raf; RafC)	Pan	NK156-5	P04049	RAF1	4479	731	16	3621	566	16	-19	0.026
TRPV4	Pan	NN464-2	Q9HBA0	TRPV4	915	90	10	741	60	8	-19	0.016
MEKK2 (MAP3K2)	Pan	NK108-5	Q9Y2U5	MAP3K2	24101	303	1	19506	362	2	-19	0.010
STAT1	Pan+ S727	PN667	P42224	STAT1	11021	382	3	8946	321	4	-19	0.017
HRAS (H-Ras)	Y157	PN755	P01112	HRAS	6258	166	3	5086	273	5	-19	0.009
Met (HGF receptor)	Pan	NK110-2	P08581	MET	19637	743	4	15992	337	2	-19	0.027
ATG2A	Pan	NN209-1	Q2TAZ0	ATG2A	1806	179	10	1471	145	10	-19	0.028
FOXO1A (FKHR; FKHRL1)	S256	11115	Q12778	FOXO1	725	97	13	592	58	10	-18	0.040
PRKAR2A (PRKAR2; PKR2)	Pan	sc-137220	P13861	PRKAR2A	20	3	16	17	4	24	-18	0.038
TRIM28 (TIF1B)	S473	PK833	Q13263	TRIM28	838	169	20	693	152	22	-17	0.015
KCNQ2	Pan	NN275-1	O43526	KCNQ2	514	61	12	425	52	12	-17	0.022

14-3-3 (KCIP-1)	Pan	sc-1657	P31946	YWHAB	968	82	8	808	61	7	-17	0.029
Plk1 (PLK)	Pan	NK145-2	P53350	PLK1	28422	514	2	23786	866	4	-16	0.012
CDC34	Pan	C25820 610250	P49427	CDC34	5955	427	7	4989	274	6	-16	0.016
HRAS (H-Ras)	Pan	NN281-3	P01112	HRAS	6418	586	9	5410	509	9	-16	0.045
ATM	Pan	NK230-1	Q13315	ATM	15436	902	6	13015	987	8	-16	0.002
CDK15 (PFTAIRE2; ALS2CR7)	Pan	NK004-2	Q96Q40	CDK15	8166	171	2	6892	174	3	-16	0.006
ADRA2C (ADRA2L2; ADRA2RL2)	Pan	NN190-2	P18825	ADRA2C	212	107	50	180	99	55	-15	0.034
DLG4 (PSD95)	Pan	NN142	P78352	DLG4	9953	1528	15	8471	1219	14	-15	0.042
NRP1	Pan	NN604-2	O14786	NRP1	20438	1125	6	17403	643	4	-15	0.013
Hsc70 (HSPA8; Hsc70; HSP73; HSPA10)	Pan	NN060-12	P11142	HSPA8	1769	71	4	1511	110	7	-15	0.034
MEKK1 (MAP3K1)	Pan	KAP-SA001	Q13233	MAP3K1	2784	383	14	2382	454	19	-14	0.033
PML	Pan+ S518	PN641	P29590	PML	12768	110	1	10932	287	3	-14	0.015
RIOK2	S332+ S335+ S337	PK890	Q9BVS4	RIOK2	11389	756	7	9766	439	4	-14	0.019
IKZF1	Y413	PN707	Q13422	IKZF1	642	59	9	556	42	8	-13	0.027
Met (HGF receptor)	Pan	NK110-4	P08581	MET	10649	265	2	9226	543	6	-13	0.025
CAMK2d	Pan	NK019-3	Q13557	CAMK2D	13916	237	2	12127	298	2	-13	0.016
RSK1 (RPS6KA1; p90RSK)	S221	PK804	Q15418	RPS6KA1	2242	251	11	1954	321	16	-13	0.050
PPP3CC (Calcinerin Ag)	Pan+ S463	PP506	P48454	PPP3CC	4161	784	19	3629	678	19	-13	0.024
ERK1 (MAPK3; ERT2)	S283	PK879	P27361	MAPK3	18022	354	2	15718	949	6	-13	0.044
SRPK1	S222	PK819	Q96SB4	SRPK1	392	49	13	342	47	14	-13	0.001
Huntingtin (HTT)	Pan+ S417+ S419	PN828	P42858	HTT	4394	146	3	3842	38	1	-13	0.040
CDK15 (PFTAIRE2; ALS2CR7)	Pan	NK004-3	Q96Q40	CDK15	6910	406	6	6080	249	4	-12	0.035
Tyro3	Y685+ Y686	PK848	Q06418	TYRO3	609	56	9	536	42	8	-12	0.025
CD63	Pan	ab68418	P08962	CD63	9594	494	5	8494	702	8	-11	0.018
MEKK6 (MAP3K6; ASK2)	Pan	NK225-2	O95382	MAP3K6	5787	372	6	5137	457	9	-11	0.049
ATR	Pan	NK237-1	Q13535	ATR	8122	220	3	7240	183	3	-11	0.012
MLK4 (MAP3K21)	Pan	NK280-1	Q5TCX8	MAP3K21	11348	136	1	10170	196	2	-10	0.018

p70S6K (S6Ka; RPS6KB1)	Pan	NK223-4	P23443	RPS6KB1	10262	812	8	9208	630	7	-10	0.027
CDK1 (CDC2)	T161	11134	P06493	CDK1	5966	344	6	5425	212	4	-9	0.042
UT-A1 (Slc14a2; HUT2; UT2)	Pan	NN355-1	Q15849	SLC14A2	342	3	1	311	9	3	-9	0.028
DNAPK (PRKDC)	Pan	NK048-6	P78527	PRKDC	27666	1476	5	25164	988	4	-9	0.040
CDK5	Y15	PK570	Q00535	CDK5	8322	240	3	7582	243	3	-9	0.023
Cas-L	Pan+ Y166	PN505	Q14511	NEDD9	9706	525	5	8867	611	7	-9	0.006
CDK1 (CDC2)	Pan	sc-954	P06493	CDK1	11465	227	2	10489	329	3	-9	0.037
CDK6	Pan	C150M	Q00534	CDK6	9012	375	4	8284	530	6	-8	0.025
ILK1 (ILK)	Pan	KAP-ST203	Q13418	ILK	225	8	4	207	4	2	-8	0.031
NFKB1	S903	PN634	P19838	NFKB1	18069	559	3	16636	160	1	-8	0.042
ERK1 (MAPK3; ERT2)	Pan	NK055-2	P27361	MAPK3	6499	246	4	5984	393	7	-8	0.046
MKK3 (MAP2K3; MEK3)	Pan	NK101-6	P46734	MAP2K3	18500	73	0	17095	45	0	-8	0.001
Raf1 (c-Raf; RafC)	Pan	NK155-5	P04049	RAF1	8251	743	9	7653	678	9	-7	0.035
CDK7 (MO15)	Pan	NK030-1	P50613	CDK7	6901	470	7	6413	573	9	-7	0.024
ErbB2 (Neu; HER2)	Y1248	PK613	P04626	ERBB2	9188	417	5	8540	303	4	-7	0.020
AMPKa2 (PRKAA2)	S377	PK522	P54646	PRKAA2	6756	188	3	6291	198	3	-7	0.028
PRMT5	T634	PN549	O14744	PRMT5	21174	422	2	20052	738	4	-5	0.041
DNMT3A	S105	PN746	Q9Y6K1	DNMT3A	16328	276	2	15547	58	0	-5	0.041
GTF2I	S412	PN602	P78347	GTF2I	10249	459	4	9917	420	4	-3	0.047
TP53 (p53)	S6+S9	PN637	P04637	TP53	19420	566	3	18849	623	3	-3	0.032
CDK1 (CDC2)	T161	PK561	P06493	CDK1	5565	425	8	5408	395	7	-3	0.018
CDC25B	Pan	NP002-4	P30305	CDC25B	11204	158	1	10926	130	1	-2	0.006
Fyn	Pan	NK065-2	P06241	FYN	6521	112	2	6401	92	1	-2	0.017
MRLC1	T19+S20	PN836	P24844	MYL9	15972	386	2	16236	349	2	2	0.020
IRS1	Y612	44-816G	P35568	IRS1	423	31	7	433	34	8	2	0.050
NOS3 (eNOS)	T1175+ S1177	PN712	P29474	NOS3	19919	540	3	20431	453	2	3	0.030
FOXO3 (FKHRL1)	S253	PN821	O43524	FOXO3	11084	150	1	11504	199	2	4	0.032
GABBR1 (GABA B Receptor 1; GPRC3A)	T873	PN796	Q9UBS5	GABBR1	8232	166	2	8556	120	1	4	0.029
PTPRK (PTP-kappa)	Y916	PP524	Q15262	PTPRK	15708	348	2	16527	510	3	5	0.029
GCN2 (EIF2AK4)	Pan	AP8062a	Q9P2K8	EIF2AK4	85	8	9	89	8	9	5	0.023

ERK5 (MAPK7; BMK)	Pan	sc-398015	Q13164	MAPK7	9154	493	5	9678	373	4	6	0.026
Met (HGF receptor)	Pan	NK110-3	P08581	MET	2163	112	5	2327	154	7	8	0.034
PPP5C (PP5C; PP5; PPT)	Y119	PP507	P53041	PPP5C	13138	1194	9	14258	1204	8	9	0.016
EGFR (ErbB1)	Y1172	XBP-4085	P00533	EGFR	2840	50	2	3090	103	3	9	0.022
HMGCR	S872	PN705	P04035	HMGCR	9269	500	5	10108	371	4	9	0.019
PTPN1 (PTP1B)	Y46	PP533	P18031	PTPN1	14053	341	2	15347	674	4	9	0.040
IKKb (IkBKB; IKKB)	Pan	KAP-TF118	O14920	IKBKB	3043	68	2	3324	96	3	9	0.016
CDK11B (PITSLRE; p58/GTA; CLK-1)	Pan	sc-928	P21127	CDK11B	2497	169	7	2736	163	6	10	0.044
ZAP70	Pan	Z24820 610240	P43403	ZAP70	122	7	5	134	3	2	10	0.042
MEK5 (MAP2K5; MKK5)	S311	PK699	Q13163	MAP2K5	14896	619	4	16430	586	4	10	0.002
Mos	Pan	NK112	P00540	MOS	5186	219	4	5722	72	1	10	0.049
GIT1	Y545	PN517	Q9Y2X7	GIT1	2208	165	7	2436	187	8	10	0.039
p73 (TP73)	Y99	PN861	O15350	TP73	5285	130	2	5849	94	2	11	0.035
PU.1	S146	PN647	P17947	SPI1	1781	106	6	1976	161	8	11	0.038
FAK (PTK2)	S722	sc-16662-R	Q05397	PTK2	3661	419	11	4064	482	12	11	0.035
GLUT2 (SLC2A2)	Pan	NN262-1	P11168	SLC2A2	2520	155	6	2800	198	7	11	0.017
PPP2R4 (PP2A subunit B'; PTPA)	Y223	PP550	Q15257	PTPA	10131	96	1	11281	357	3	11	0.042
PPPM1B (PP2Cb; PPM1B)	Y367	PP540	O75688	PPM1B	4732	402	9	5271	559	11	11	0.046
ACTB (beta-actin)	Y53	PN501	P60709	ACTB	3865	212	5	4357	89	2	13	0.036
RSK1 (RPS6KA1; p90RSK)	S380	PK805	Q15418	RPS6KA1	1520	176	12	1714	145	8	13	0.013
PCTK2 (PCTAIRE2; CDK17))	S180	PK756	Q00537	CDK17	5889	803	14	6646	825	12	13	0.016
CLK3	Pan	AP7531a	P49761	CLK3	36	11	29	41	9	23	13	0.039
Tec	Y519	PK829	P42680	TEC	847	38	5	960	61	6	13	0.041
PAK2 (PAKg)	Pan	sc-1872	Q13177	PAK2	2865	153	5	3255	119	4	14	0.030
TERF1	T371	PN675	P54274	TERF1	1211	169	14	1380	131	10	14	0.025
Nek2	Pan	71-3700	P51955	NEK2	375	18	5	428	31	7	14	0.036
Gab1	Y406	PN516	Q13480	GAB1	1956	344	18	2238	430	19	14	0.043
ATR	S435+ S436	PK528	Q13535	ATR	14817	1393	9	16973	1108	7	15	0.024
Nek2	S171	PK732	P51955	NEK2	13503	703	5	15538	1042	7	15	0.017

UGDH	Y352	PN782	O60701	UGDH	2910	238	8	3348	139	4	15	0.031
SGK1	Pan	NK294-1	O00141	SGK1	752	67	9	876	81	9	16	0.008
Met (HGF receptor)	S1236	PK705	P08581	MET	3216	86	3	3761	164	4	17	0.049
MAFG	Pan+ S124	PN617	O15525	MAFG	3341	153	5	3931	216	5	18	0.012
FLT3 (STK1; CD135)	Pan	NK240-2	P36888	FLT3	5109	60	1	6012	156	3	18	0.006
IkBa (MAD3; IkBa)	Pan	DB075	P25963	NFKBIA	1290	40	3	1527	54	4	18	0.036
KRS2	Pan	33-3000	Q13043	STK4	3980	286	7	4715	381	8	18	0.021
MOR1 (mu opiod receptor)	T372+ S377+ T378	PN802	P35372	OPRM1	4856	252	5	5786	88	2	19	0.020
HIPK2	Pan	NK272-1	Q9H2X6	HIPK2	2872	229	8	3427	318	9	19	0.045
ARID1A	S363	PN740	O14497	ARID1A	9211	866	9	11025	546	5	20	0.026
BLNK	Y84	44-220	Q8WV28	BLNK	4451	749	17	5334	485	9	20	0.043
ERK1 (MAPK3; ERT2)	Pan	NK055-4	P27361	MAPK3	2085	71	3	2524	63	2	21	0.002
PKN1 (PRK1)	T774	PK781	Q16512	PKN1	3972	629	16	4813	407	8	21	0.034
p38a MAPK (MAPK14; CSBP; MXI2; SAPK2a)	Pan	NK120-4	Q16539	MAPK14	1021	134	13	1259	185	15	23	0.031
GSK3b	Pan	KAP-ST002	P49841	GSK3B	2105	182	9	2602	196	8	24	0.000
NMDAR2A NMDA (GRIN2A; Glutamate [NMDA] receptor subunit epsilon-1)	Pan	NN297-1	Q12879	GRIN2A	673	90	13	835	87	10	24	0.001
VACAMKL	Y245	PK892	Q8NCB2	CAMKV	541	45	8	671	71	11	24	0.020
SMARCA4	S610+ S613	PN726	P51532	SMARCA4	1067	146	14	1327	96	7	24	0.019
GATA3	S369	PN702	P23771	GATA3	1914	141	7	2416	213	9	26	0.012
PTPN12 (PTP-PEST; PTPG1)	Pan	P105M	Q05209	PTPN12	158	39	24	201	39	19	27	0.015
Nur77	Pan+ S351	PN636	P22736	NR4A1	2995	71	2	3835	195	5	28	0.033
VEGFR2 (KDR)	Y1059	11531	P35968	KDR	186	20	11	242	33	13	30	0.030
TrkB (NTRK2)	Y706+ Y707	PK917	Q16620	NTRK2	1708	65	4	2247	100	4	32	0.039
AMPKa1 (PRKAA1)	T183+ S184	PK521	Q13131	PRKAA1	4926	499	10	6566	564	9	33	0.039
HGK (MAP4K4; NIK; ZC1)	Pan	NK300-1	O95819	MAP4K4	295	16	5	393	34	9	34	0.019
MEK5 (MAP2K5; MKK5)	Pan	sc-1287	Q13163	MAP2K5	2772	339	12	3752	156	4	35	0.031

JAK1	Pan	sc-277	P23458	JAK1	259	13	5	356	11	3	37	0.028
NLK	Pan	sc-8211	Q9UBE8	NLK	317	45	14	447	30	7	41	0.013
Ankyrin (ANK1; Ankyrin-R)	Pan	NN195-1	P16157	ANK1	482	59	12	683	94	14	42	0.043
WNK4 (PRKWNK4)	Pan	NK255-1	Q96J92	WNK4	1563	241	15	2373	362	15	52	0.034
TAO1 (TAOK1)	S181	PK826	Q7L7X3	TAOK1	262	17	6	419	50	12	60	0.023
GRK1 (Rhodopsin kinase)	Pan	sc-8004	Q15835	GRK1	288	20	7	615	49	8	114	0.017

Supplementary Table S3. Phosphoproteins that significantly changed between Controls and AD cases. Only proteins detected with phosphosite-specific antibodies were included. Average signal intensity and standard deviation were calculated for the 3 batches of Controls (C1-C3) or the 3 batches of AD cases (AD1-AD3). The changes in signal intensity between Controls and AD cases are presented as the percentage change from Controls (%CFC). Signal decrease in AD cases is represented in blue, and the signal increase is represented in light pink. Signal intensities obtained for each target between 3 batches of age-matched Controls and 3 batches of AD cases were compared using a paired t-test.

					Controls			AD				
**Target Name**	**Antibody P-Site**	**Cat No.**	**UniProt ID**	**Gene name**	**Mean**	**SD**	**SD %**	**Mean**	**SD**	**SD %**	**%CFC**	**p-Value**
RPS6	S235+S236	PN685	P62753	RPS6	3221	994	31	2116	650	31	-34	0.049
TARDBP	S409+S410	PN674	Q13148	TARDBP	1994	259	13	1424	160	11	-29	0.030
MERTK (MER)	Y753	PK704	Q12866	MERTK	1919	141	7	1400	84	6	-27	0.028
TrkA (NGFR; NTRK1)	T496	sc-8058	P04629	NTRK1	35	5	14	26	3	12	-27	0.024
HGS (Hrs)	Y216	PN519	O14964	HGS	3876	260	7	2893	66	2	-25	0.049
EphB2	Y780	PK610	P29323	EPHB2	3076	403	13	2298	330	14	-25	0.015
VIM (Vimentin)	S34	KAM-CC246	P08670	VIM	388	30	8	294	41	14	-24	0.019
Huntingtin (HTT)	S421	PN829	P42858	HTT	3191	378	12	2413	355	15	-24	0.009
ERF	T526	PN584	P50548	ERF	7199	526	7	5558	391	7	-23	0.011
TAO1 (TAOK1)	Y309	PK827	Q7L7X3	TAOK1	2974	522	18	2307	363	16	-22	0.031
IRF3	T135	PN610	Q14653	IRF3	3146	84	3	2476	178	7	-21	0.043
HRAS (H-Ras)	Y157	PN755	P01112	HRAS	6258	166	3	5086	273	5	-19	0.009
FOXO1A (FKHR; FKHRL1)	S256	11115	Q12778	FOXO1	725	97	13	592	58	10	-18	0.040
TRIM28 (TIF1B)	S473	PK833	Q13263	TRIM28	838	169	20	693	152	22	-17	0.015
RIOK2	S332+S335+ S337	PK890	Q9BVS4	RIOK2	11389	756	7	9766	439	4	-14	0.019
IKZF1	Y413	PN707	Q13422	IKZF1	642	59	9	556	42	8	-13	0.027
RSK1 (RPS6KA1; p90RSK)	S221	PK804	Q15418	RPS6KA1	2242	251	11	1954	321	16	-13	0.050
ERK1 (MAPK3; ERT2)	S283	PK879	P27361	MAPK3	18022	354	2	15718	949	6	-13	0.044
SRPK1	S222	PK819	Q96SB4	SRPK1	392	49	13	342	47	14	-13	0.001
Tyro3	Y685+Y686	PK848	Q06418	TYRO3	609	56	9	536	42	8	-12	0.025

CDK1 (CDC2)	T161	11134	P06493	CDK1	5966	344	6	5425	212	4	-9	0.042
CDK5	Y15	PK570	Q00535	CDK5	8322	240	3	7582	243	3	-9	0.023
NFKB1	S903	PN634	P19838	NFKB1	18069	559	3	16636	160	1	-8	0.042
ErbB2 (Neu; HER2)	Y1248	PK613	P04626	ERBB2	9188	417	5	8540	303	4	-7	0.020
AMPKa2 (PRKAA2)	S377	PK522	P54646	PRKAA2	6756	188	3	6291	198	3	-7	0.028
PRMT5	T634	PN549	O14744	PRMT5	21174	422	2	20052	738	4	-5	0.041
DNMT3A	S105	PN746	Q9Y6K1	DNMT3A	16328	276	2	15547	58	0	-5	0.041
GTF2I	S412	PN602	P78347	GTF2I	10249	459	4	9917	420	4	-3	0.047
TP53 (p53)	S6+S9	PN637	P04637	TP53	19420	566	3	18849	623	3	-3	0.032
CDK1 (CDC2)	T161	PK561	P06493	CDK1	5565	425	8	5408	395	7	-3	0.018
MRLC1	T19+S20	PN836	P24844	MYL9	15972	386	2	16236	349	2	2	0.020
IRS1	Y612	44-816G	P35568	IRS1	423	31	7	433	34	8	2	0.050
NOS3 (eNOS)	T1175+ S1177	PN712	P29474	NOS3	19919	540	3	20431	453	2	3	0.030
FOXO3 (FKHRL1)	S253	PN821	O43524	FOXO3	11084	150	1	11504	199	2	4	0.032
GABBR1 (GABA B Receptor 1; GPRC3A)	T873	PN796	Q9UBS5	GABBR1	8232	166	2	8556	120	1	4	0.029
PTPRK (PTP-kappa)	Y916	PP524	Q15262	PTPRK	15708	348	2	16527	510	3	5	0.029
PPP5C (PP5C; PP5; PPT)	Y119	PP507	P53041	PPP5C	13138	1194	9	14258	1204	8	9	0.016
EGFR (ErbB1)	Y1172	XBP-4085	P00533	EGFR	2840	50	2	3090	103	3	9	0.022
HMGCR	S872	PN705	P04035	HMGCR	9269	500	5	10108	371	4	9	0.019
PTPN1 (PTP1B)	Y46	PP533	P18031	PTPN1	14053	341	2	15347	674	4	9	0.040
MEK5 (MAP2K5; MKK5)	S311	PK699	Q13163	MAP2K5	14896	619	4	16430	586	4	10	0.002
GIT1	Y545	PN517	Q9Y2X7	GIT1	2208	165	7	2436	187	8	10	0.039
p73 (TP73)	Y99	PN861	O15350	TP73	5285	130	2	5849	94	2	11	0.035
PU.1	S146	PN647	P17947	SPI1	1781	106	6	1976	161	8	11	0.038
FAK (PTK2)	S722	sc-16662-R	Q05397	PTK2	3661	419	11	4064	482	12	11	0.035
PPP2R4 (PP2A	Y223	PP550	Q15257	PTPA	10131	96	1	11281	357	3	11	0.042

subunit B'; PTPA)													
PPPM1B (PP2Cb; PPM1B)	Y367	PP540	O75688	PPM1B	4732	402	9	5271	559	11	11	0.046
ACTB (beta-actin)	Y53	PN501	P60709	ACTB	3865	212	5	4357	89	2	13	0.036
RSK1 (RPS6KA1; p90RSK)	S380	PK805	Q15418	RPS6KA1	1520	176	12	1714	145	8	13	0.013
PCTK2 (PCTAIRE 2; CDK17))	S180	PK756	Q00537	CDK17	5889	803	14	6646	825	12	13	0.016
Tec	Y519	PK829	P42680	TEC	847	38	5	960	61	6	13	0.041
TERF1	T371	PN675	P54274	TERF1	1211	169	14	1380	131	10	14	0.025
Gab1	Y406	PN516	Q13480	GAB1	1956	344	18	2238	430	19	14	0.043
ATR	S435+S436	PK528	Q13535	ATR	14817	1393	9	16973	1108	7	15	0.024
Nek2	S171	PK732	P51955	NEK2	13503	703	5	15538	1042	7	15	0.017
UGDH	Y352	PN782	O60701	UGDH	2910	238	8	3348	139	4	15	0.031
Met (HGF receptor)	S1236	PK705	P08581	MET	3216	86	3	3761	164	4	17	0.049
MOR1 (mu opiod receptor)	T372+S377+T378	PN802	P35372	OPRM1	4856	252	5	5786	88	2	19	0.020
ARID1A	S363	PN740	O14497	ARID1A	9211	866	9	11025	546	5	20	0.026
BLNK	Y84	44-220	Q8WV28	BLNK	4451	749	17	5334	485	9	20	0.043
PKN1 (PRK1)	T774	PK781	Q16512	PKN1	3972	629	16	4813	407	8	21	0.034
VACAMKL	Y245	PK892	Q8NCB2	CAMKV	541	45	8	671	71	11	24	0.020
SMARCA4	S610+S613	PN726	P51532	SMARCA4	1067	146	14	1327	96	7	24	0.019
GATA3	S369	PN702	P23771	GATA3	1914	141	7	2416	213	9	26	0.012
VEGFR2 (KDR)	Y1059	11531	P35968	KDR	186	20	11	242	33	13	30	0.030
TrkB (NTRK2)	Y706+Y707	PK917	Q16620	NTRK2	1708	65	4	2247	100	4	32	0.039
AMPKa1 (PRKAA1)	T183+S184	PK521	Q13131	PRKAA1	4926	499	10	6566	564	9	33	0.039
TAO1 (TAOK1)	S181	PK826	Q7L7X3	TAOK1	262	17	6	419	50	12	60	0.023
